# Control of Tissue Fibrosis by 5-Methoxytryptophan, an Innate Anti-Inflammatory Metabolite

**DOI:** 10.3389/fphar.2021.759199

**Published:** 2021-11-11

**Authors:** Kenneth K Wu

**Affiliations:** ^1^ Institute of Cellular and System Medicine, National Health Research Institutes, Zhunan, Taiwan; ^2^ Institute of Biotechnology, College of Life Science, National Tsing-Hua University, Hsinchu, Taiwan

**Keywords:** fibrosis, 5-methoxytryptophan, liver cirrhosis, heart failure, fibroblasts, myofibroblasts, hepatic stellate cells

## Abstract

Tissue fibrosis causes debilitating human diseases such as liver cirrhosis, heart failure, chronic kidney disease and pulmonary insufficiency. It is a dynamic process orchestrated by specific subsets of monocyte-macrophages, fibroblasts, pericytes and hepatic stellate cells. Fibrosis is linked to tissue inflammation. Pro-inflammatory macrophages promote fibrosis by driving myofibroblast differentiation and macrophage myofibroblast transition. Myofibroblasts express α-smooth muscle cell actin (α-SMA) and secrete extracellular matrix (ECM) proteins notably collagen I and III. Deposition of ECM proteins at injury sites and interstitial tissues distorts normal structure and impairs vital functions. Despite advances in the mechanisms of fibrosis at cellular, molecular and genetic levels, prevention and treatment of fibrotic diseases remain poorly developed. Recent reports suggest that 5-methoxytryptophan (5-MTP) is effective in attenuating injury-induced liver, kidney, cardiac and pulmonary fibrosis. It inhibits macrophage activation and blocks fibroblast differentiation to myofibroblasts. Furthermore, it inhibits hepatic stellate cell differentiation into myofibroblasts. As 5-MTP is an endogenous molecule derived from tryptophan catabolism via tryptophan hydroxylase pathway, it is well-suited as a lead compound for developing new anti-fibrotic drugs. This article provides an overview of 5-MTP synthesis, and a critical review of its anti-fibrotic activities. Its mechanisms of actions and potential therapeutic value will be discussed.

## Introduction

Fibrous deposition at the injured tissue is a fundamental repair process (Eming et al., 2014). However, uncontrolled fibrous deposition at the injured and normal interstitial tissues leads to structural remodeling and functional defects (Henderson et al., 2020). Fibrosis of diverse human organs including liver, heart, kidney and lung causes debilitating diseases with considerable morbidity and mortality. Fibrosis was previously considered to be a passive event. Recent studies with cellular tracking, gene expression profiling and single cell analysis shed lights on the dynamic nature of fibrosis (Wynn and Vannella, 2016). Tissue injury by diverse insults leads to recruitment of pro-inflammatory and pro-fibrotic cells to the injury sites where they undergo phenotypic, transcriptional and metabolic changes to promote fibroblast differentiation into myofibroblasts (Gibb et al., 2020). Myofibroblasts express α-smooth muscle actin (α-SMA) and secrete extracellular matrix (ECM) proteins notably collagen I and III and fibronectin (Gibb et al., 2020). ECM proteins deposit at the injured sites and the adjacent interstitial space and distort normal structure and impair organ function. Extensive investigations of the pathological features and molecular mechanisms of fibrosis have shown that despite distinct structural and functional characteristic of the vital organs, fibrosis of liver, heart, kidney and lung shares common pathological, cellular and molecular features. Inflammation is a common hallmark of fibrotic disorders. In fact, fibrosis occurs in inflammatory microenvironment which is enriched with macrophage infiltration (Misharin et al., 2017). Macrophages release myriad pro-inflammatory cytokines and chemokines to elicit inflammatory responses and cause tissue damage. Furthermore, they crosstalk with fibroblasts and promote fibroblasts differentiation to myofibroblasts (Karlmark et al., 2009; Murray et al., 2011; Tang et al., 2019; Zhang F. et al., 2020). Macrophages may be transdifferentiated into myofibroblasts through macrophage myofibroblast transition (MMT) (Murray et al., 2011).

Under normal conditions, resident fibroblasts are quiescent. Upon activation by factors released during tissue injury, they undergo phenotypic switch accompanied by metabolic and transcriptional reprogramming (Gibb et al., 2020). They are the major source of myofibroblasts and the key effector of organ fibrosis. Advances in single cell analytic techniques coupled with RNA sequencing and transcriptomes, have shed lights on heterogeneity of resident fibroblasts in various organs and the involvement of a subset of fibroblasts destined to be differentiated into myofibroblasts (Dobie et al., 2019; Krenkel et al., 2019; Ramachandran et al., 2019; Reyfman et al., 2019). Recent studies using single cell analytic techniques to explore the fibroblast mystery identities a common principle of pathological fibrosis: activation of a selective group of fibroblasts which undergo progressive transcriptional reprogramming and step-wise differentiation into myofibroblasts to generate fibrosis.

**FIGURE 1:**
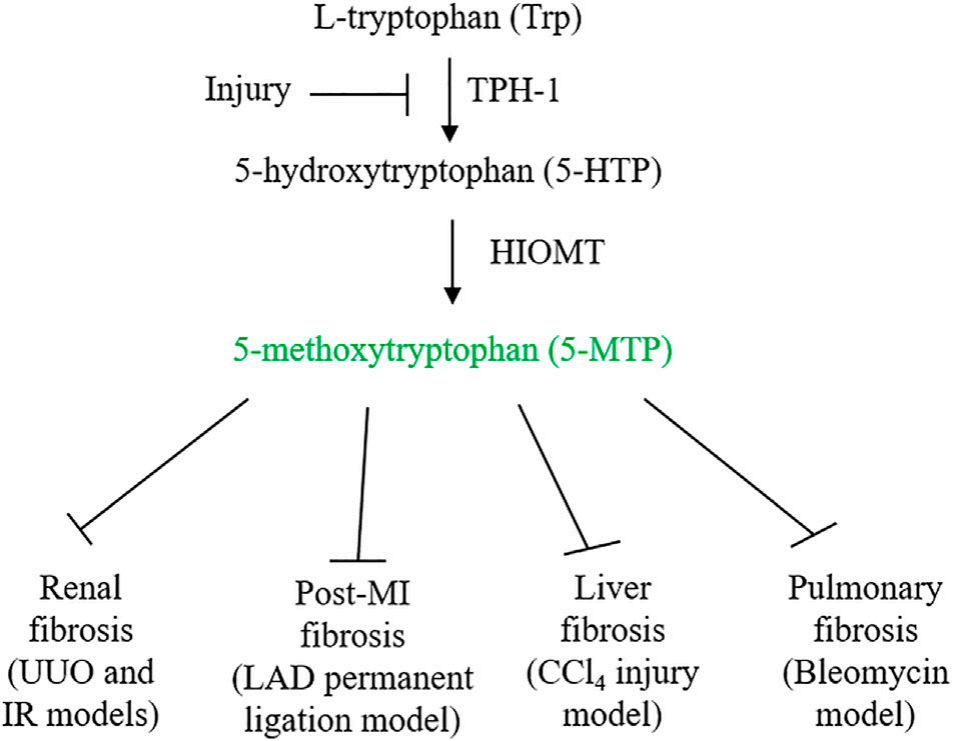
5-MTP synthetic pathway and its inhibition by injury. Ischemia and inflammatory mediators suppress 5-MTP production by downregulating TPH-1 expression. 5-MTP suppression contributes to tissue inflammation and fibrosis. Administration of 5-MTP was reported to control fibrosis in vital organs.

Despite molecular and cellular advances in the understanding of fibrosis, there is a gap in prevention and treatment of this devastating human disorder. Several recent reports suggest that 5-methoxytryptophan (5-MTP) is effective in attenuating cardiac, renal, hepatic and pulmonary fibrosis in animal models (Figure 1). 5-MTP was discovered in Wu’s laboratory as a cyclooxygenase-2 (COX-2) suppressing factor (Deng et al., 2002). It was originally named cytoguardin as it was thought to protect tissues from inflammatory damage (Deng et al., 2002; Wu, 2021). Metabolomic analysis coupled with genetic approaches identified cytoguardin as a tryptophan (Trp) metabolite, 5-MTP (Cheng et al., 2012). 5-MTP was shown to be derived from the tryptophan hydroxylase (TPH) pathway (Cheng et al., 2012). It is produced in fibroblasts, vascular endothelial cells (ECs) and smooth muscle cells, renal and bronchial epithelial cells (Wang et al., 2016). Vascular EC releases 5-MTP into the extracellular milieu via Golgi vesicular trafficking (Wang et al., 2016), and is a major cellular source of blood 5-MTP. 5-MTP was reported to protect endothelial barrier function, control endothelial expression of adhesion molecules and inhibit monocyte/macrophage transmigration (Chu et al., 2016; Wang et al., 2016). Furthermore, 5-MTP inhibits macrophage activation and blocks macrophage release of pro-inflammatory cytokines and chemokines and expression of COX-2 (Wang et al., 2016). These findings indicate that 5-MTP is an innate anti-inflammatory molecule (Wu et al., 2020). As vascular EC is a major cellular source of circulating 5-MTP, 5-MTP is considered to be a new endothelial arsenal against inflammation (Durán and Sánchez, 2016). In view of its efficacy in controlling myofibroblast differentiation and pathological fibrosis in vital organs, 5-MTP has the potential to fulfill the therapeutic gap. The purpose of this paper is to review the anti-fibrotic and anti-inflammatory actions of 5-MTP and comment on the mechanisms of actions and the use of 5-MTP as a lead compound in developing new anti-fibrotic drugs.

## 5-MTP Biosynthesis Is Catalyzed by TPH-1 and Hydroxyindole O-Methyltransferase (HIOMT)

5-MTP is produced from Trp via two enzymatic steps: TPH catalyzes the conversion of Trp to 5-hydroxytryptophan (5-HTP) and HIOMT, conversion of 5-HTP to 5-MTP (Cheng et al., 2012) (Figure 1). With respect to TPH, two isoforms were identified and characterized (Boularand et al., 1990; Walther et al., 2003). TPH-2 is expressed in neuronal and pineal cells while TPH-1 is expressed in peripheral tissues. 5-MTP producing cells such as fibroblasts and vascular endothelial cells express only TPH-1. As knockdown of TPH-1 with siRNA abrogates 5-MTP production in fibroblasts, TPH-1 is the isoform catalyzing 5-MTP production (Cheng et al., 2012). Examination of TPH-1 deleted mice has linked TPH-1 to cardiac function: genetic deletion of TPH-1 leads to cardiac failure in mice (Côté et al., 2003). Polymorphism of *TPH-1* was identified in humans which was considered to be linked to cardiac dysfunction (Lai et al., 2005). However, there has not been reports directly linking TPH-1 polymorphism to cardiac fibrosis or functional impairment. By contrast, it has been reported that TPH-1 expression is suppressed by pro-inflammatory cytokines which results in reduced 5-MTP production and endothelial dysfunction (Wang et al., 2016; Chu et al., 2016). Furthermore, TPH-1 expression was suppressed in ischemic cardiac and renal tissues which was associated with decreased 5-MTP level, cardiac inflammation and fibrosis and functional impairment (Chen CH. et al., 2019; Hsu et al., 2021) (Figure 1). Thus, TPH-1 defends against inflammation, fibrosis and tissue damage via 5-MTP production. HIOMT was originally detected in pineal cells as the final enzymatic step in melatonin synthesis (Axelrod and Weissbach, 1960, 1961). As it catalyzes conversion of N-acetylserotonin to N-acetyl-5-methoxytryptamine (melatonin), it is also called acetylserotonin O-methyltransferase (ASMT). ASMT is a single gene product but three mRNA isoforms due to alternative splicing were detected in pineal cells (Rodriquez et al., 1994; Donohue et al., 1993). Isoform 345 was reported to be the active isoform in melatonin synthesis (Botros et al., 2013). 5-MTP producing cells express only ASMT298 isoform which was reported to be catalytically active in 5-MTP synthesis (Chen et al., 2018). *ASMT* contains polymorphism in the promoter region which affects ASMT expression and was reported to be a risk factor of autism spectrum disorders (Melke et al., 2008). Several exon mutations affecting ASMT activity were reported which may be associated with sleep disorder (Pagan et al., 2011). Association of ASMT polymorphism/mutation with tissue fibrosis has not been reported. Further work to characterize the relationship may yield valuable information regarding the roles of TPH-1 and HIOMT in inflammation and fibrosis.

## 5-MTP Controls Systemic Inflammation

5-MTP defends against systemic inflammation as reported in a LPS-induced sepsis murine model (Wang et al., 2016). Mice receiving LPS injection develop symptoms and signs mimicking human sepsis. They exhibit surge of circulating cytokines and chemokines (“cytokine storm”), which is accompanied by macrophage infiltration in multiple organs. The infiltrated macrophages express COX-2 and inducible nitric oxide synthase (iNOS) and release abundant cytokines and chemokines. Activated macrophages are responsible not only for organ inflammation but also for cytokine storm. Wang et al. were the first to report that intraperitoneal administration of 5-MTP blocks macrophage expression of COX-2 and release of cytokines in LPS-treated mice. It attenuates macrophage accumulation in lungs and reduces inflammatory response in spleen (Wang et al., 2016). Importantly, it ameliorates sepsis manifestations and reduces sepsis-mediated mortality (Wang et al., 2016).

5-MTP exerts anti-inflammatory effects by targeting activated macrophages. Furthermore, *in vitro* and *in vivo* studies suggest that 5-MTP controls macrophage activation by inhibiting p38 MAPK-mediated NF-κB activation (Wang et al., 2016). It has also been reported that 5-MTP inhibits transcription co-activator p300 binding and histone acetyltransferase (HAT) activity (Chen et al., 2012; Wang et al., 2016). p300 is a master mediator of pro-inflammatory gene expression (Grunstein, 1997; Goodman and Smolik, 2000; Thompson et al., 2004). It binds to promoter-bound transactivators and bridges this message with transcriptional machinery (Grunstein, 1997; Goodman and Smolik, 2000; Thompson et al., 2004). p300 HAT acetylates histone to open up chromatin structure thereby facilitating transactivator binding (Grunstein, 1997; Goodman and Smolik, 2000; Thompson et al., 2004). It acetylates a large group of transactivators to promote their transcriptional activation of pro-inflammatory genes including COX-2, iNOS and pro-inflammatory cytokines and chemokines (Deng et al., 2003; Deng et al., 2004). Thus, by interfering with p300 binding and p300 HAT activity, 5-MTP is effective in inhibiting the activity of key transactivators such as NF-κB, C/EBPβ, AP-1 and CREB and attenuating expression of pro-inflammatory genes mediated by those transactivators.

## 5-MTP Protects Against Post-Infarct Myocardial Fibrosis

**FIGURE 2:**
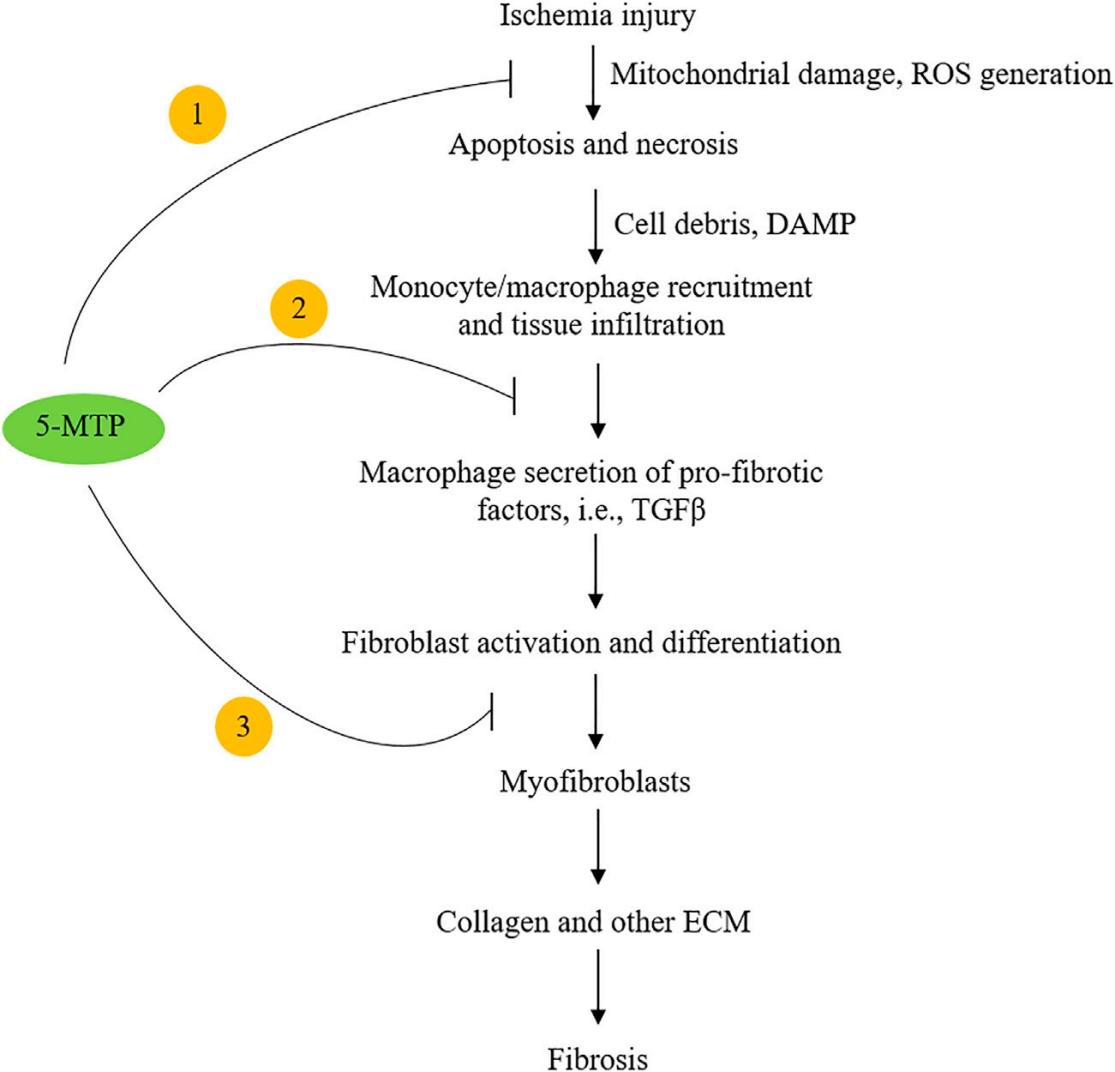
Highlight of the potential mechanisms by which 5-MTP inhibits cardiac fibrosis. 5-MTP exerts cellular actions by ① scavenging ROS and reducing apoptosis; ② blocking macrophage secretion of TGFβ and other pro-fibrotic factors and ③ reducing myofibroblasts.

Following coronary artery occlusion, ischemia and/or ischemia-reperfusion injury induces cardiomyocyte necrosis and apoptosis (Cheng et al., 1996; Olivetti et al., 1996). The damaged cells release chemotactic factors such as CXCL2 and CCL2 which recruit blood monocytes to the injured sites (Deshmane et al., 2009; Guo et al., 2020). Monocytes are differentiated into macrophages, which along with resident macrophages infiltrate the injured and adjacent normal tissues and elicit inflammatory responses. They crosstalk with fibroblasts and induce myofibroblast differentiation. Myofibroblasts drive cardiac fibrosis by release of collagen and an array of extracellular matrix proteins (van den Borne et al., 2010; Kramann et al., 2013; Duffield et al., 2013). Macrophage-mediated inflammatory tissue damage and myocardial fibrosis are cardinal pathophysiological processes causing post-MI myocardial structural changes, functional impairment and heart failure (Prabhu and Frangogiannis, 2016; Talman and Ruskoaho, 2016; Kingery et al., 2017; Bernard et al., 2018). Hsu et al. have recently reported that intraperitoneal administration of 5-MTP in a left anterior descending artery occlusion (LAD) rat model (De Villiers and Riley, 2020) reduces macrophage infiltration, attenuates myocardial fibrosis and preserves ventricular structure and function (Hsu et al., 2021). They first determined route, timing and doses of 5-MTP administration, and found that administration of two doses of 5-MTP at 17 mg/kg within 24 h after LAD occlusion optimally reduced inflammation and fibrosis and preserved structural integrity and ventricular function (Hsu et al., 2021). Additional dosing after the 24 h-window did not provide additional advantage. At 48 h after LAD occlusion, there was a significant reduction of macrophages in the infarct and peri-infarct areas in 5-MTP-treated rats when compared to control rats (Hsu et al., 2021). Furthermore, expression of IL-1β, IL-6, IL-18, CCL2, CXCL2 and CXCL10 was significantly lower in 5-MTP treated than in control rats (Hsu et al., 2021). At day 28 after LAD occlusion, extensive transmural fibrosis and interstitial fibrosis developed which was accompanied by structural remodeling. Intraperitoneal 5-MTP administration within 24 h of LAD occlusion attenuated cardiomyocyte apoptosis and infarct volume and improved left ventricular function accompanied by reduced fibrosis and transmural scar in the left ventricular region. TGFβ, a master mediator of fibrosis (Leask and Abraham, 2004; Bernard et al., 2018) was increased in infarct zone as a result of macrophage infiltration. Macrophage iNOS expression which was increased in the infarct zone has been implicated in myocardial fibrosis and heart failure (Chen CH. et al., 2019). 5-MTP was shown to reduce TGFβ level and attenuate iNOS expression in macrophages at the infarct zone (Hsu et al., 2021). These findings suggest that 5-MTP prevents post-MI myocardial fibrosis by controlling ROS generation, inhibiting macrophage activation and suppressing pro-fibrotic gene expression (Figure 2).

## 5-MTP Defends Kidneys Against Urethral Obstruction-Induced Fibrosis

Metabolomic analysis of serum metabolites in chronic kidney diseases identified 5-MTP as a major metabolite which is inversely associated with progression of kidney diseases (Chen DQ. et al., 2019). In experimental animal models such as unilateral urethral obstruction (UUO) murine model, renal production of 5-MTP was reported to be reduced accompanied by progressive renal damage leading to renal fibrosis and functional impairment. Urethral obstruction elicits renal inflammation and inflammation-mediated tissue damage and fibrosis. It has been shown that renal tubular epithelial cells produce 5-MTP (Wang et al., 2016). It is possible that decrease of renal 5-MTP production following UUO is due to suppression of renal tubular cell TPH-1. Chen et al. reported that intraperitoneal administration of 5-MTP rescues kidney from UUO-induced renal inflammation and fibrosis (Chen CH. et al., 2019). 5-MTP administration reduced macrophage infiltration and pro-inflammatory gene expression. 5-MTP downregulated expression of pro-fibrotic genes suggesting that 5-MTP prevents myofibroblast differentiation in injured kidneys. *In vitro* epithelial cell experiments reveal that TGFβ and LPS induce expression of pro-fibrotic factors accompanied by suppression of TPH-1 expression and 5-MTP production. Pretreatment with 5-MTP rescues renal epithelial cells from TGF-β- and LPS-induced pro-inflammatory and pro-fibrotic phenotypic switch. Furthermore, 5-MTP blocks NF-κB activation induced by LPS. TPH-1 overexpression in renal epithelial cells restores 5-MTP production and renders epithelial cells resistant to LPS- and TGFβ-induced pro-fibrotic and pro-inflammatory changes. Renal ischemia-reperfusion injury results in chronic inflammatory and profibrotic pathophysiological changes resembling ischemia-reperfusion injury to the heart. 5-MTP attenuates ischemia-reperfusion induced renal inflammation and fibrosis in a fashion similar to protection of heart from inflammatory tissue damage and fibrosis (Chen DQ. et al., 2019).

Following UUO or ischemia-reperfusion injury, several cell types were considered to be the source of myofibroblasts (Duffield, 2014; Wouters et al., 2015). Recent studies using single cell RNA sequencing and spatial transcriptomics, have shown that myofibroblasts are derived from resident fibroblasts and pericytes (Kuppe et al., 2021). It remains to be investigated whether 5-MTP has a direct effect on the fibrogenic cells.

## 5-MTP Suppresses Hepatic Stellate Cell Activation and Transdifferentiation

Uncontrolled liver fibrosis leads to debilitating liver cirrhosis and liver failure. Liver fibrosis is a common pathological event induced by viral infections, chronic alcoholism, non-alcoholic liver disease and non-alcoholic steatohepatitis. These pathophysiological conditions are associated with release of myriad factors to activate HSCs (Tsuchida and Friedman, 2017). HSCs are localized at the junction of sinusoid endothelial cells and hepatocytes. They represent a major cell type in liver: approximately 10% of liver cells are HSCs. At resting state, they are quiescent. Upon liver injury, damaged hepatocytes release pro-fibrotic factors and generate ROS and damage-associated molecular patterns (DAMP), which activate HSCs and induce their differentiation into myofibroblasts (Du et al., 2018; Khomich et al., 2019; An et al., 2020). It has been shown that activated HSCs exhibit major changes in energy metabolism shifting from oxidative phosphorylation to aerobic glycolysis and glutaminolysis (Du et al., 2018; Khomich et al., 2019). Mitochondrial TCA cycle is interrupted and metabolites such as succinate are accumulated and released. Extracellular succinate induces HSC differentiation through binding to a specific succinate receptor, SUCNR-1 (also known as GPR91) (Li et al., 2015; Li et al., 2016; Cho, 2018).

**FIGURE 3:**
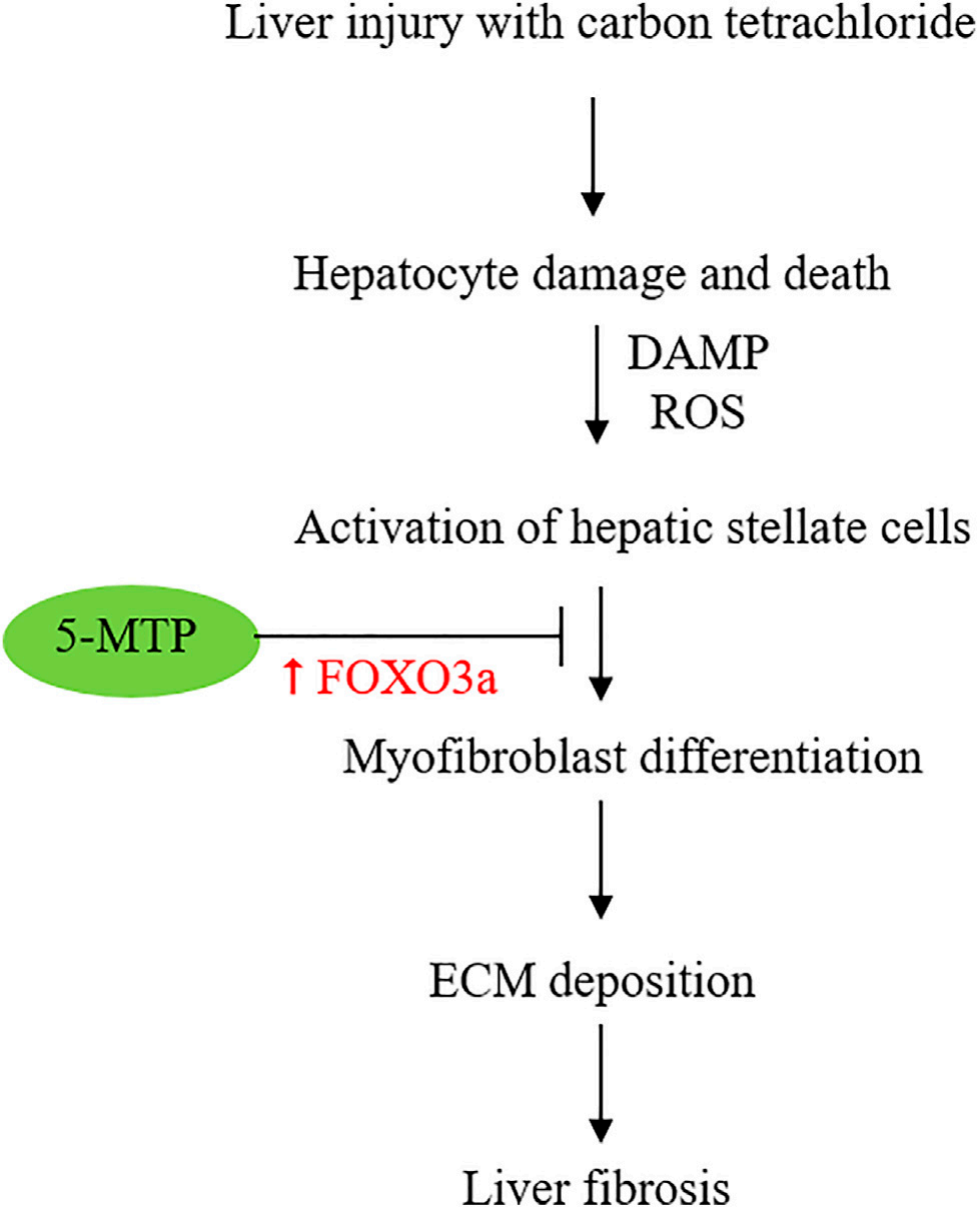
Schematic illustration of the effect of 5-MTP on attenuating HSC differentiation to myofibroblasts via FOXO3a upregulation. FOXO3a was reported to suppress miR21 expression and thereby restore ATG5 and ATG5-mediated autophagy.

5-MTP was reported to attenuate CCl4-induced liver cirrhosis in a murine model. 5-MTP at a relatively low dose (5 mg/kg twice weekly for 8 weeks) was effective in reducing α-SMA expressing cells and attenuating liver fibrosis (Tong et al., 2021). In the *in vitro* experiments with cultured HSC, LX-2 cells, 5-MTP suppressed TGFβ-induced expression α-SMA, fibronectin, collagen I and III, suggesting that 5-MTP may block differentiation of HSCs into myofibroblasts. Tong et al. have shown that 5-MTP blocks the pro-fibrotic property of HSC by regulating FOXO3a/miR21/ATG5 pathway (Tong et al., 2021). They have provided experimental evidence that miR21 binds and inhibits ATG5 and thereby controls autophagy. FOXO3a inhibits miR21 expression and restores ATG5 for participation in autophagy. Autophagy was reported to reduce hepatic fibrosis (Zhu et al., 1999; Hidvegi et al., 2010). However, other reports suggest autophagy activates HSC and promotes hepatic fibrosis (Hernández-Gea et al., 2012). The complex role of autophagy in hepatic fibrosis may be due to different cell types, stimuli and experimental conditions (Mao and Fan, 2015; Li et al., 2020). By using genetic knockdown and pharmacological inhibition, Tong et al have shown that 5-MTP inhibits HSC proliferation and differentiation by upregulating FOXO3a and restoration of autophagy. This observation was consistent with previous reports that 5-MTP prevents mesenchymal stromal cell (MSC) senescence by upregulation of FOXO3 (Chang et al., 2017) and that FOXO3 plays a pivotal role in controlling fibrosis (Al-Tamari et al., 2018). Taken together, the reported findings suggest that 5-MTP attenuates liver fibrosis by upregulating FOXO3a (Figure 3) and the consequent suppression of miR21 expression. It is to be noted that effective 5-MTP concentrations to prevent MSC senescence are one to two orders of magnitude higher than those used in blocking HSC activation and differentiation. Further studies are needed to clarify the dosing difference.

## 5-MTP Inhibits Bleomycin-Induced Pulmonary Fibrosis

Pulmonary alveolar epithelial cells are vulnerable to injury by environmental toxins, drugs, radiation and immune attacks. Uncontrolled damage leads to cell loss and fibrosis (Noble et al., 2012; Wuyts et al., 2013). Pulmonary fibrosis destroys normal alveolar structure and impairs lung function resulting in chronic debilitating illness (Sgalla et al., 2019). Recent advances in the cellular and molecular mechanisms of lung fibrosis provide evidence to indicate that pulmonary fibrosis shares with fibrosis of other organs key pathological features such as recruitment of monocytes to promote fibrosis, conversion of resident fibroblasts to myofibroblasts and activation of pro-fibrotic transcriptional programs (Zepp et al., 2017; Peyser et al., 2019). It is thus not unexpected that 5-MTP inhibits bleomycin-induced pulmonary fibrosis in a murine model (Fang et al., 2020). In this model, Fang et al. showed that 5-MTP administration to bleomycin-treated mice reduced collagen deposition, myofibroblasts accumulation and alveolar architectural destruction. It disrupted TGFβ/SMAD3 and PI-3K/Akt pathways. *In vitro* experiments provide evidence to support the observation that 5-MTP inhibits fibroblast differentiation to myofibroblasts and reduces fibroblast migration by blocking TGFβ signaling pathway (Fang et al., 2020). These findings suggest that 5-MTP is effective in attenuating pulmonary fibrosis due to external injury. It is unclear whether 5-MTP exerts a similar effect on idiopathic pulmonary fibrosis, the most common and the most severe form of human pulmonary interstitial fibrotic diseases (Martinez et al., 2005).

## Potential Mechanisms by Which 5-MTP Controls Fibrosis

**FIGURE 4:**
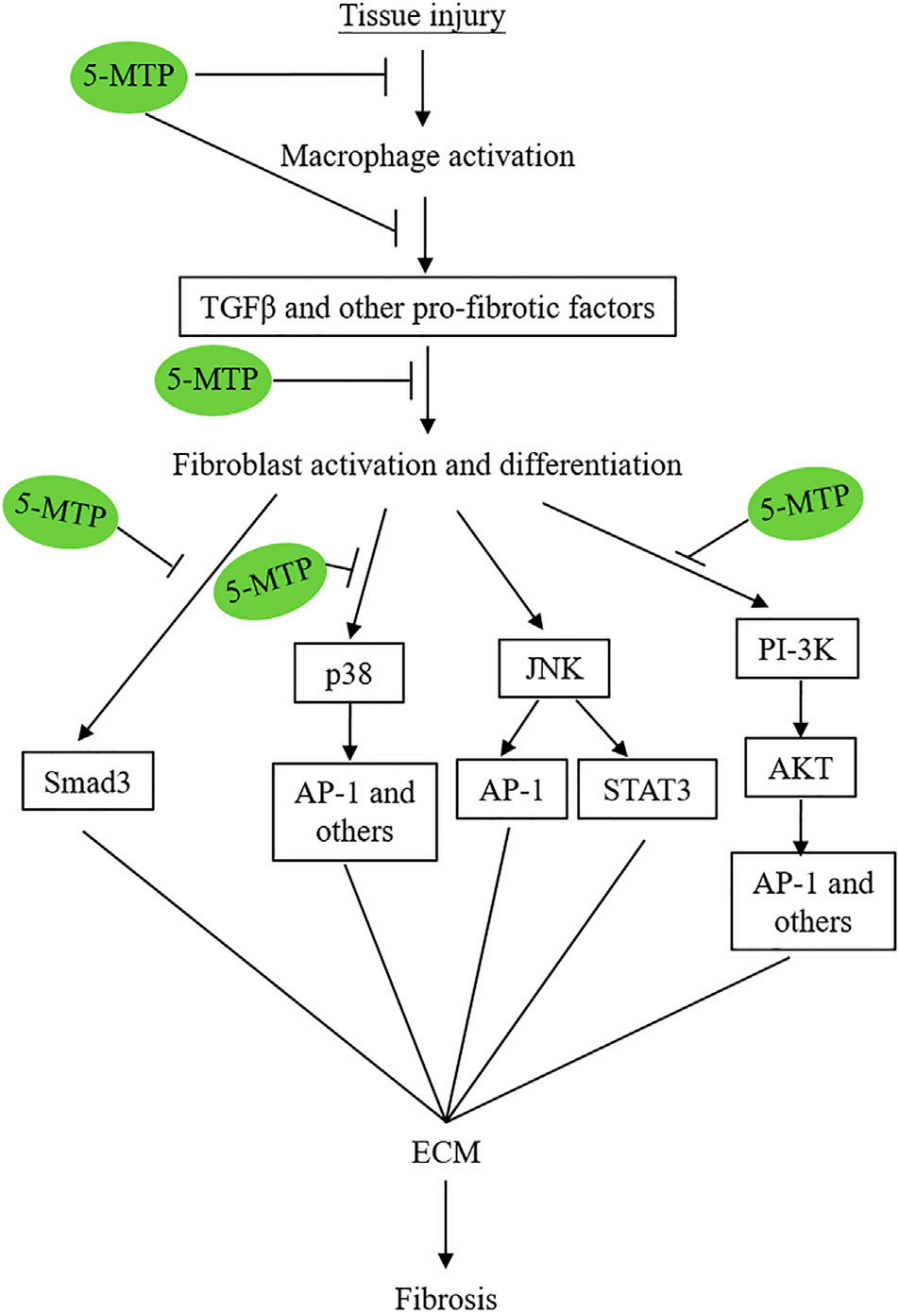
Simplified graphic scheme illustrating the 5-MTP targets via which it attenuates tissue-injury-induced tissue fibrosis.

The exact mechanisms by which 5-MTP reduces fibrosis in multiple organs are not completely understood and remain to be investigated. However, recent reports suggest that 5-MTP exerts its anti-fibrotic actions by controlling multiple steps of pro-fibrotic cellular changes, transcriptional reprogramming and signaling pathways (Figure 4).

### 5-MTP Inhibits Ischemia-Induced Apoptosis

Immediately following coronary artery occlusion, hypoxia induces rapid metabolic changes. Glycolysis is enhanced while oxidative phosphorylation is shut down, resulting in TCA cycle interruption and accumulation of succinate (Chouchani et al., 2014; Zhang et al., 2018). Furthermore, mitochondrial electron transport chain (ETC) becomes disorganized with a reverse electron transport resulting in generation of reactive oxygen species (ROS) (Chouchani et al., 2014). Balance of mitochondrial dynamics (mitochondrial fusion vs. fission) is altered to favor mitochondrial fission resulting in mitochondrial fragmentation which is accompanied by uncontrolled mitochondrial membrane permeability resulting in cellular necrosis and apoptosis (Ong et al., 2010; Ramachandra et al., 2020). Massive cardiomyocyte death creates an infarct zone. Damaged and dead heart cells produce chemotactic factors to recruit neutrophils, monocytes and lymphocytes from circulating blood to the infarct and peri-infarct zone. DAMP as well as cell debris and mitochondrial DNA is generated to activate the recruited blood cells and elicit inflammatory responses (Ramachandran et al., 2012; Mouton et al., 2018). Excessive inflammation induces additional cardiac cell damage and expand the infarct size and triggers the subsequent tissue fibrosis. Reported data indicate that 5-MTP attenuates apoptosis and reduces infarct size following LAD occlusion in the rat model (Hsu et al., 2021). 5-MTP reduces caspase 3 level of myocardial tissues and caspase 3-positive cardiomyocytes at the peri-infarct region (Hsu et al., 2021). These findings suggest that 5-MTP reduces mitochondrial damage and controls mitochondrial release of cytochrome C. Furthermore, 5-MTP preserved TOM20, a mitochondrial outer membrane protein, which is decreased by ischemia, suggesting that 5-MTP maintains mitochondrial membrane integrity during ischemic attacks and thus attenuates apoptosis via the mitochondrial-dependent pathway (Hsu et al., 2021). Inhibition of ischemia- and toxin-induced mitochondrial damage and the consequent release of pro-apoptotic factors from mitochondria is a major attribute by which 5-MTP attenuates inflammation and fibrosis. This event represents an early window of opportunity for fibrosis control.

### 5-MTP Scavenges ROS and Prevents ROS-Induced Cell Death and Senescence

During tissue injury by ischemia, toxins and chemicals, there is continued generation of ROS: initially via damaged mitochondria followed by activation of cytosolic NADPH oxidase and xanthine oxidase (Chouchani et al., 2016; Zhang Y. et al., 2020). ROS are scavenged by mitochondrial antioxidant enzymes notably MnSOD and peroxiredoxins (Prdx) (Cox et al., 2009) and cytosolic antioxidant enzymes such as catalase. ROS induce a broad spectrum of pathological changes including cell deaths, inflammation and fibrosis (Gonzalez-Gonzalez et al., 2017; Moris et al., 2017). It was reported that ROS promote fibrosis by enhancing TGFβ signaling (Gonzalez-Gonzalez et al., 2017). 5-MTP was reported to reduce ROS generation in mesenchymal stromal cells induced by high glucose by upregulating MnSOD and catalase activities (Chang et al., 2017). Hsu et al. reported in the rat MI model that 5-MTP administration within 24 h after LAD occlusion reduced accumulation of ROS (Hsu et al., 2021). Their results reveal that 5-MTP restored MnSOD and Prdx3 and inhibited NADPH oxidase by suppressing expression of NOX2 and NOX4 (Hsu et al., 2021).

### 5-MTP Controls the Transcriptional Network That Drives Fibrosis

The subset of fibroblasts, which are destined to be differentiated to myofibroblasts exhibit distinct transcriptional network. Several transcriptional activators have been identified as master driver of fibroblast commitment. PU.1 was reported to drive fibroblast polarization and to be essential for tissue fibrosis (Wohlfahrt et al., 2019). Inhibition of PU.1 prevents bleomycin-induced skin fibrosis. AP-1 transactivator plays a pivotal role in organ fibrosis. Activation of c-Jun, a subunit of AP-1, was sufficient to induce diverse fibrotic diseases (Wernig et al., 2017; Schulien et al., 2019). AP-1 binds to the promoter region of profibrotic and pro-inflammatory genes including collagen and ECM proteins, growth factors, cytokines and COX-2 (Manabe et al., 2002), which contribute to cardiac and pulmonary fibrosis (Rajasekaran et al., 2013). Other transactivators such as NF-κB, C/EBPβ and CREB were reported to be activated in fibrotic tissues and contribute to fibrosis. For example, NF-κB has been shown to regulate activation of HSCs and hepatic myofibroblast differentiation (Luedde and Schwabe, 2011) and promote cardiac remodeling (Hamid et al., 2011). C/EBPβ phosphorylation at Thr 217 contributes to lung fibrosis in mice (Buck and Chojkier, 2011) and mesenchymal specific deletion of C/EBPβ suppresses pulmonary fibrosis (Hu et al., 2012).

5-MTP was discovered as a suppressor of COX-2 expression (Deng et al., 2002; Cheng et al., 2012). It inhibits COX-2 transcription by inhibiting binding of NF-κB, AP-1, C/EBPβ and CREB to COX-2 promoter (Chen et al., 2012). As COX-2 is considered to be a prototypic immediate early gene and shares with pro-inflammatory cytokines, adhesion molecules and pro-fibrotic genes common promoter characteristics, it is likely that 5-MTP attenuates injury-induced organ fibrosis through blocking activation and binding of AP-1 and other transactivators to the promoter regions of pro-fibrotic genes. p300 inhibitor was reported to ameliorate cardiac and renal fibrosis (Rai et al., 2017) indicating that p300 is intimately involved in pro-fibrotic transcriptional program. 5-MTP inhibits p300 binding and HAT activity via which it exerts potent actions on controlling transcription of pro-inflammatory and pro-fibrotic genes.

### 5-MTP Disrupts Pro-Fibrotic Signaling Pathways

A number of signaling pathways including p38 MAPK (Ma et al., 1999; Stambe et al., 2004; Molkentin et al., 2017; Turner and Blythe, 2019), TGFβ/SMAD3 (Fang et al., 2020), PI3K/AKT (Zhang et al., 2016; Hsu et al., 2017; Fang et al., 2020; Hu et al., 2020); JNK (Grynberg et al., 2017) and STAT3 (Chakraborty et al., 2017) were reported to mediate tissue fibrosis. p38 MAPK plays a central regulatory role. Molkentin et al. have used transgenic approaches to demonstrate that fibroblast-specific transgenic overexpression of MAP kinase 6 (MKK6), an upstream inducer of p38 MAPK, results in interstitial and perivascular fibrosis in the heart, lung and kidney accompanied by increased myofibroblast (Molkentin et al., 2017). Genetic deletion of Mapk14 (coding for p38 MAPK) blocks fibroblast differentiation to myofibroblasts in a murine cardiac injury model (Ma et al., 1999). These results suggest that p38 MAPK is a major signaling molecule of fibroblast differentiation and activation and plays a central role in in pathological fibrosis.

TGFβ induces fibroblast activation and myofibroblast differentiation (Froese et al., 2016; Meng et al., 2016) by binding to a membrane heterodimeric receptor which activate and differentiate fibroblasts via several signaling pathways (Luo and Lodish, 1996). The canonical pathway is mediated via SMAD3 which has been shown to be critical for myofibroblast differentiation (Hu et al., 2007; Dobaczewski et al., 2010). TGFβ may induce fibroblast activation and myofibroblast differentiation via other signaling pathways independent of SMAD3 among which p38 MAPK pathway and PI-3K/AKT are well characterized non-canonical signaling pathways. Furthermore, it has been reported that p38 MAPK mediates fibrogenic signal through SMAD3 photophosphorylation suggesting a crosstalk between p38 MAPK and SMAD (Furukawa et al., 2003). It has been suggested that various signaling pathways converge at STAT-3 to mediate TGFβ-induced tissue fibrosis (Chakraborty et al., 2017).

5-MTP was reported to block p38 MAPK activation in a number cellular models under different stresses. For example, it was reported that 5-MTP protects endothelial VE-cadherin and therefore endothelial barrier function by blocking p38 MAPK activation (Chu et al., 2016). 5-MTP suppresses cytokine-induced vascular SMC phenotypic switch by blocking p38 MAPK and thereby maintaining contractile vascular SMC contractile phenotype (Chen CH. et al., 2019). Importantly, 5-MTP inhibits macrophage activation and secretion of cytokines by inhibiting p38 MAPK signaling pathway (Wang et al., 2016). An additional supportive evidence is derived from cancer cell epithelial mesenchymal transition (EMT) (Cheng et al., 2016). EMT is an important cellular phenotypic switch via which cancer cells achieve metastatic characteristics. It was reported that 5-MTP was effective in reducing A549 cancer cell EMT via suppressing p38 MAPK (Cheng et al., 2016). Taken together, the reported data suggest that 5-MTP exerts its multiple protective effects through blocking p38 MAPK activation and p38 MAPK signaling pathways.

Fang et al. reported that 5-MTP attenuates pulmonary fibrosis by blocking TGFβ-induced SMAD3 and PI3K/AKT signaling pathways (Fang et al., 2020). Thus, 5-MTP controls tissue fibrosis by inhibiting several pro-fibrotic signaling pathways (Figure 4). Of note, these signaling pathways cross-talk to promote fibroblast activation, differentiation and fibrosis.

It remains to be determined how 5-MTP blocks diverse signaling pathways. Preliminary data suggest that 5-MTP acts via a specific membrane receptor (Wang et al., 2018). It is possible that 5-MTP blocks p38 MAPK, SMAD3 and PI-3K/AKT through cross-talk between the signaling pathway mediated by 5-MTP receptor activation and stress-induced pro-fibrotic signaling pathways. As 5-MTP receptors have not been identified and characterized, the potential cross-talk is hypothetical and requires further validation.

## Conclusion

Vascular endothelial cells, renal and pulmonary epithelial cells, fibroblasts and cardiomyocytes produce 5-MTP which defends vital organs against injury and maintains tissue homeostasis. Pro-inflammatory mediators suppress 5-MTP production through inhibiting TPH-1 expression, and disrupting the homeostatic balance leading to tissue damage and fibrosis. Supplementation with exogenous 5-MTP rescues tissues from inflammatory damage and prevents fibrosis and structural remodeling. 5-MTP was recently reported to protect against post-infarct cardiac fibrosis and left ventricular remodeling in a rat LAD permanent ligation model (Hsu et al., 2021). The experimental data suggest that 5-MTP exerts anti-fibrotic effects through control of early ROS accumulation, apoptosis and macrophage recruitment. 5-MTP attenuates renal fibrosis and functional failure in a UUO model (Chen DQ. et al., 2019). 5-MTP reduces bleomycin-induced alveolar epithelial cell damage and induces interstitial fibrosis through downregulating TGFβ and PI-3K signaling pathways (Fang et al., 2020). Finally, 5-MTP was reported to alleviate CCl_4_-induced liver fibrosis through FoxO3a-mediated autophagy (Tong et al., 2021). These reports indicate that 5-MTP possesses universal anti-fibrotic actions and is a potential lead compound for developing new therapy for fibrotic disorder.

5-MTP exerts the anti-fibrotic effect by inhibiting macrophage recruitment and activation, which is closely linked to its anti-inflammatory actions. Macrophage infiltration in injured tissues is a cardinal manifestation of tissue inflammation, fibrosis and organ failure. 5-MTP blocks monocyte transmigration and macrophage secretion of chemokines thereby reducing macrophage accumulation at the tissue injured site. Furthermore, 5-MTP inhibits macrophage release of pro-inflammatory cytokines notably IL-1β, TNFα and IL-6 and pro-fibrotic factors such as TGFβ, PDGF and cytokines. Macrophages are heterogenous. Subsets of macrophages are functionally selective for inflammatory responses and fibrotic formation, respectively. It remains to be determined whether 5-MTP possesses selective actions on functionally distinct subsets of macrophages.

5-MTP controls macrophage activation by inhibiting p300 HAT activity and binding of pro-inflammatory transactivators such as NF-κB, AP-1, C/EBPβ and CREB. Since AP-1 plays a central role in mediating fibrosis, it is likely that 5-MTP inhibits pro-fibrotic gene expressions in fibroblasts and myofibroblasts by blocking the activity and binding of AP-1. p38 MAPK is a key signaling pathway mediating inflammation and fibrosis. 5-MTP is known to inhibit p38 MAPK activation in macrophages, vascular ECs and SMCs. It is likely that 5-MTP inhibits pro-inflammatory transactivators by disrupting p38 MAPK activation and downstream signaling.

The antifibrotic and anti-inflammatory actions of 5-MTP are likely to be mediated by interaction between 5-MTP and its cellular surface receptors. Preliminary data suggest that the 5-MTP receptor is a G protein-coupled receptor. It is possible that signaling from 5-MTP receptors inhibits p38 MAPK activation, and/or upregulating p38 MAPK inhibitors (Kumar et al., 2003).

Development of anti-fibrotic therapy has been hampered by incomplete understanding of the complex cellular and molecular mechanisms underlying initiation and progression of fibrosis. Another hurdle that hampers development of antifibrotic therapy is lack of an effective strategy to block selectively pathological fibrosis while preserving physiological fibrosis, which is vital to tissue repair. 5-MTP represents a new class of compounds that could overcome the obstacles. It offers several advantages. Foremost is that it is an endogenously produced metabolite with known anti-inflammatory and tissue protection properties. Secondly, it suppresses but does not completely abolish macrophage activation and fibroblast differentiation. It is therefore well suited to be a lead compound for developing new anti-fibrotic drugs. 5-MTP derivatives were recently granted multi-national patents. As post-MI heart failure and post-injury chronic kidney diseases emerge as a major disease burden with great health and socioeconomic impacts, 5-MTP derivatives will be a valuable addition to prevention and treatment of those devastating illnesses.

## Abbreviations

α-SMA, α-smooth muscle actin; ECM, extracellular matrix; MMT, macrophage myofibroblast transition; 5-MTP, 5-methoxytryptophan; COX-2, cyclooxygenase-2; Trp, tryptophan; TPH, tryptophan hydroxylase; ECs, endothelial cells; 5-HTP, 5-hydroxytryptophan; ASMT, acetylserotonin O-methyltransferase; iNOS, inducible nitric oxide synthase; HAT, histone acetyltransferase; LAD, left anterior descending artery occlusion; UUO, unilateral urethral obstruction; DAMP, damage-associated molecular patterns; MSC, mesenchymal stromal cell; ETC, electron transport chain; ROS, reactive oxygen species; Prdx, peroxiredoxins; MKK6, MAP kinase 6; EMT, epithelial mesenchymal transition.
